# Geometric Accuracy of 3D-Printed Composite Dental Restorations Compared with the Original STL Design

**DOI:** 10.3390/jfb17050251

**Published:** 2026-05-19

**Authors:** Tommaso Rossi, Giulia Pascoletti, Michele Calì, Giuliana Baiamonte, Fulvia Concetta Rita Monaco, Elisabetta Maria Zanetti, Alberto Audenino, Gianpaolo Serino, Bartolomeo Coppola, Andrea Messina, Nicola Scotti

**Affiliations:** 1 Department of Surgical Sciences, Dental School Lingotto, University of Turin, 10126 Turin, Italy; tommaso.rossi@studenti.polito.it; 2 Department of Engineering, University of Perugia, Via Duranti 93, 06125 Perugia, Italy; giulia.pascoletti@unipg.it (G.P.); elisabetta.zanetti@unipg.it (E.M.Z.); 3 Electric, Electronics and Computer Engineering Department, University of Catania, 95125 Catania, Italy; michele.cali@unict.it (M.C.); giuliana.baiamonte@unict.it (G.B.); fulvia.monaco@unict.it (F.C.R.M.); 4 Department of Mechanical and Aerospace Engineering, Politecnico di Torino, 10129 Turin, Italy; alberto.audenino@polito.it (A.A.); gianpaolo.serino@polito.it (G.S.); 5 Department of Applied Science and Technology (DISAT), Politecnico di Torino, 10129 Turin, Italy; bartolomeo.coppola@polito.it (B.C.); andrea.messina@polito.it (A.M.)

**Keywords:** 3D printing, dental restorations, resin-based composites, deviation analysis geometric accuracy, marginal fit, internal gap, trueness

## Abstract

Additive manufacturing (AM) enables customized, efficient restorative workflows, though the accuracy of 3D-printed restorations may be compromised by polymerization, sintering shrinkage, and post-processing. This study evaluated the geometric accuracy of 3D-printed partial restorations compared with the computer-aided design (CAD) reference. The null hypothesis stated that no significant differences would be found between Varseo Smile Crown^plus^ (by BEGO, Italy) and IRIXMax (by DWS System, Italy) materials, which are printed and cured with different technologies. A model was prepared for an overlay and designed with a 1.5 mm uniform thickness. Restorations were produced in two groups with two different printing processes: DLP (digital light processing)-printed Varseo Smile Crown^plus^ and SLA (stereolithography)-printed IRIXMax. Six samples per group were printed at 90° orientation and scanned. Meshes were aligned to the master geometry via pre-alignment and ICP (Iterative Closest Point) registration. Deviations were quantified in CloudCompare using mean, standard deviation (SD), and 90th percentile values. IRIXMax showed the lowest deviations from the ideal geometry, while Varseo Smile Crown^plus^ exhibited greater variability. Pairwise comparisons found IRIXMax significantly more accurate than Varseo Smile Crown^plus^. Color maps confirmed material-specific deviation patterns. IRIXMax provided the highest geometric accuracy. Material-specific calibration is essential for reliable 3D-printed definitive restorations.

## 1. Introduction

Three-dimensional (3D) printing, also known as additive manufacturing, is revolutionizing restorative dentistry by enabling the fabrication of indirect restorations through fully digital workflows [1]. The integration of intraoral scanning systems, CAD software, and 3D printing technologies allows clinicians to produce highly customized restorations while reducing material waste and streamlining clinical procedures [2,3,4]. Compared with traditional subtractive computer-aided manufacturing (CAM) techniques, additive manufacturing offers greater design freedom and the potential to fabricate complex geometries layer by layer with controlled material deposition [2].

The geometric accuracy of 3D-printed restorations is a critical determinant of their clinical success. Discrepancies between the printed restoration and the original CAD file (STL format) design may compromise marginal adaptation, internal fit, and cement space distribution, which can ultimately affect adhesive sealing and biomechanical stability [5,6,7]. Even minimal inaccuracies may translate into clinical complications such as marginal leakage, secondary caries, or mechanical failures under functional loading [6].

Several factors influence the dimensional fidelity of additively manufactured restorations, including build orientation, layer thickness, light source characteristics, post-curing protocols, and material composition [8,9,10]. Vat-photopolymerization (VPP) technologies such as digital light processing (DLP) and stereolithography (SLA) are widely used in dental applications, but their differences in light delivery, polymerization kinetics, and voxel formation may impact dimensional trueness and surface resolution [9,10]. Build angle and printing strategy have also been shown to significantly affect the precision and reproducibility of printed restorations [8,10].

Among printable materials, resin-based composites reinforced with ceramic fillers have been proposed for definitive restorations [11]. These hybrid materials aim to combine the aesthetic and mechanical properties of ceramics with the stress-absorbing capacity and intraoral reparability of composites [12,13]. Compared with glass-ceramics, composite materials demonstrate lower antagonistic wear and more favorable elastic behavior, reducing stress concentration at the adhesive interface [12]. Digital light processing and stereolithography are particularly promising for producing accurate designs with a fine surface finish [1]. A tilting SLA (TSLA) 3D printer showed high marginal trueness and precision when fabricating crowns, comparable to milled zirconia and superior to milled lithium disilicate [14]. However, polymerization shrinkage during curing is an inherent limitation of methacrylate-based composites, potentially causing volumetric contraction and residual stresses [13,15]. The magnitude of these distortions depends on filler content, monomer chemistry, degree of conversion, and curing strategy [12,13,15].

Recent in vitro studies indicate that SLA-printed crowns often exhibit superior marginal fit and trueness compared with DLP-printed crowns, highlighting the influence of printing technology on geometric fidelity [16,17]. However, a comprehensive understanding of dimensional deviations in definitive 3D-printed composite restorations, including spatial deviation mapping, remains limited. There is a clear gap in the literature regarding systematic comparisons of SLA and DLP composites for single-unit restorations, particularly using high-resolution metrological approaches. Another aspect to be considered is that post-processing is always required, and this includes procedures to remove supports that are functional elements often required in 3D printing for structural needs, in case of undercuts or overhangs, or as heat dissipators; these supports are most commonly removed mechanically with the possibility of leaving some residue or creating localized damages with an impact on the geometric accuracy [18].

All that considered, being able to assess the geometric accuracy is a mandatory issue to be able to optimize the 3D printing process [19]. The geometric correctness is generally assessed by identifying “deviations” in terms of distances between the nominal or target shape and actual shapes. The nominal shape is usually defined by specific CAD drawings, while actual shapes can be detected through various metrological techniques, moving from contact ones, based on touch probe systems (Coordinate Measuring Machine—CMM), to contactless techniques [20]. The last ones can be applied to record only the outer surface of manufactures, as in the case of structured light or laser scanning [21], or both the inner and outer geometries, as for micro-Computed Tomography (micro-CT) scans [22].

Therefore, the present study aimed to compare the three-dimensional geometric accuracy of two 3D-printed composite restorative materials fabricated as overlay restorations and evaluate their deviation from the input CAD file. The null hypothesis was that no statistically significant difference would be observed between the two tested materials and the model in Standard Tessellation Language (STL).

## 2. Materials and Methods

### 2.1. Samples Preparation

An intact human upper molar, extracted for periodontal reasons, was preserved in a 0.5% cloramine T solution at 4 °C for a month after extraction. The tooth was prepared following indirect ceramic restoration guidelines: 1.5 mm occlusal reduction, 1 mm reduction at the preparation axial walls, rounded edges and a 6-degree taper [23]. The preparation design was a circumferential chamfer occlusal to the tooth survey line 4 mm above the Cemento-Enamel Junction (CEJ). The tooth was prepared by a single experienced clinician using 3.5 magnification to verify the preparation thickness using Elite HD+ silicone index (by Zhermack, Badia Polesine, Italy), a periodontal probe (CP-15) and a diamond rotary instrument (by Komet, Verona, Italy) mounted on a red ring contra-angle handpiece.

After preparation, the tooth was scanned with the Trios 3 intraoral scanner (by 3Shape, Copenhagen, Denmark) and the restoration was designed with Dental CAD (3Shape CAD, by 3Shape, Copenhagen, Denmark). The restoration was designed with a uniform thickness of 1.5 mm.

The restorations were assigned to 2 groups according to the materials (Table 1) and their associated 3D printers with which they were produced (*n* = 6 each):

G1—Varseo Smile Crown^plus^ (by BEGO, Bolzano, Italy): is a ceramic-filled resin designed using 3D printing techniques for permanent restorations such as crowns, inlays and onlays. It is a tooth-colored, light-curing, methacrylate-based hybrid composite resin containing silanized dental glass fillers. Its inorganic filler content is 30–50% by mass, dispersed in a Bis-GMA/Bis-EMA-like methacrylate resin matrix with photoinitiators such as methyl benzoylformate and TPO. It is designed for 3D printing using digital light processing (DLP) technology.

G2—IRIXMax (by DWS System, Thiene, Italy): is a photosensitive nanoceramic composite material developed for dental applications. Designed for 3D printing using stereolithography (SLA) technology, IRIXMax enables the creation of high-precision permanent restorations such as single crowns, bridges with up to three elements, inlays, onlays and veneers. IRIXMax is a biocompatible, light-curing translucent hybrid composite material containing 42% ceramic filler by weight, dispersed in a photosensitive resin matrix.

**Figure 1:**
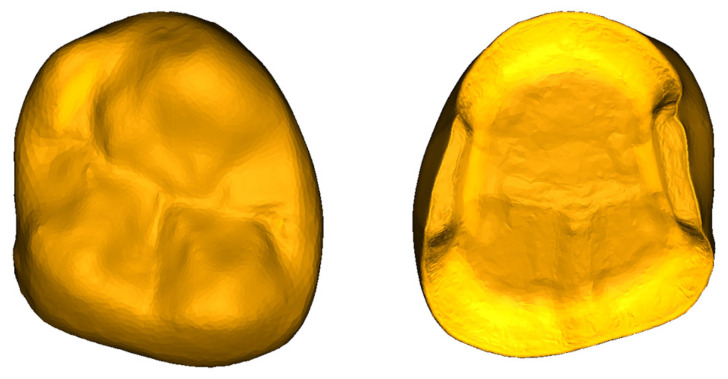
Reference model for all printed overlays.

The input geometry for all restorations was the same, called ‘master’ in the following Figure 1; the G-code files to define 3D printing trajectories were obtained from this reference geometry.

All restorations were 3D printed, using 2 different printers as follows:

G1 samples were produced by the Pro S printer (by Sprintray, Weiterstadt, Germany). It is a DLP-based 3D printer with a 405 nm LED projector. The layer thickness was 50 µm.

G2 samples were produced by DFAB Desktop (by DWS System, Thiene, Italy). It is an SLA 3D printer. The layer thickness was 50 μm.

For each group, 6 samples were produced by placing the printing support on the mesial side (90° of orientation).

After 3D printing, samples were post-processed following the manufacturer’s instructions.

Regarding G1 samples, they were washed with 96% ethanol in the dedicated disposal through immersion and shaking for 3 min to remove the uncured resin. Then samples were dried using compressed air, and the support was removed by specific side cutters. After that, the samples were post-cured for 3.42 min by ProCure 2 (by SprintRay, Germany) with a 385 nm wavelength and a patented light motion drive UV LED system.

Regarding G2 samples, they were washed with 96% ethanol in the dedicated disposal through immersion and shaking for 3 min to remove the uncured resin. Then samples were dried using compressed air, and the support was removed by specific side cutters. After that, the samples were post-cured for 9 min in the DCure photopolymerizer (by DWS Systems, Thiene, Italy).

### 2.2. Geometry Acquisition

Printed samples were digitized making use of an intraoral 3D scanner, Trios 3 (by 3shape, Denmark), to acquire occlusal and vestibular surface geometry; according to [24], this scanner’s precision is equal to 1.9 μm, while its trueness is equal to 16.8 ± 3.8 μm SD. The meshes were saved as .stl files, with nodes ranging between 5383 and 6509.

### 2.3. Statistical Analysis of Geometric Deviations

**Figure 2:**
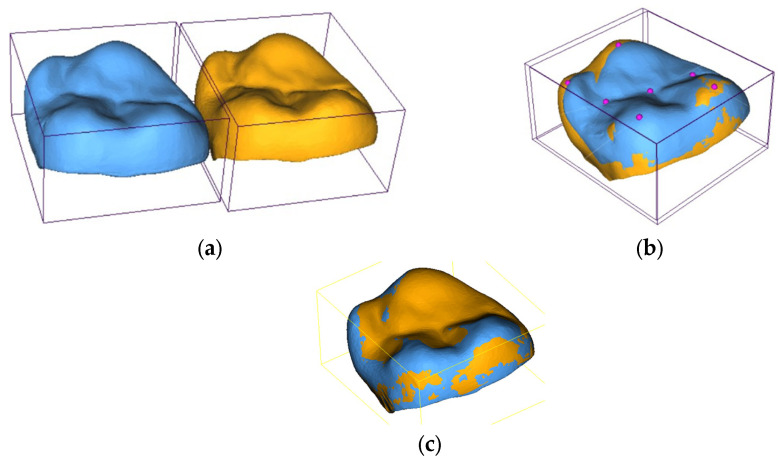
(**a**) Pre-alignment between digitized samples (blue one) and reference geometry (orange one); (**b**) alignment through landmarkers; (**c**) fine alignment through ICP algorithm.

Geometric deviation analysis was carried out using the CloudCompare open-source software (v. 2.13.2). Initially, all digitized samples were manually centered and coarsely aligned with the reference geometry (the ‘Master’), as shown in Figure 2a. This pre-alignment ensured that the sample overlay was centered and approximately aligned with the reference one, facilitating the following fine registration phase. Subsequently, a fine registration was performed using the Iterative Closest Point (ICP) algorithm (Figure 2b), which iteratively minimized the root mean square (RMS) distance between the sample and reference surfaces (Figure 2c). The optimization criterion was the minimization of the average squared error, computed as the distance between each node of the reference mesh and the nearest point on the digitized geometry. The ICP iterations were run until convergence to a root mean square (RMS) error between 0.01 mm and 0.015 mm, ensuring the accurate superimposition.

Following registration, geometric deviations between the sample and the master were quantified using the “Compute cloud/mesh distance” tool. This functionality compares each point in the aligned sample to the closest triangle on the reference mesh, generating a scalar field of deviations and enabling both statistical analysis and spatial mapping of local discrepancies. The cumulative frequency histograms of geometric deviation were calculated, both signed (where a positive value corresponds to a gap and a negative value corresponds to an interference) and unsigned. The unsigned geometric deviations were further elaborated, calculating, for each mesh, statistical outputs such as the average value, its standard deviation, and the 90th percentile.

Moreover, additional analyses were carried out to evaluate the presence of statistically significant differences among the tested materials. A pairwise comparison analysis was performed to detect the respective statistical significance. The comparison was performed using Kolmogorov–Smirnov tests, applied to the sample distributions of mean, standard deviation (SD), maximum, and 90th percentile values of geometric deviations. The significance threshold was set at *p* < 0.05.

## 3. Results

### 3.1. Descriptive Statistics of Geometric Deviations

**Figure 3:**
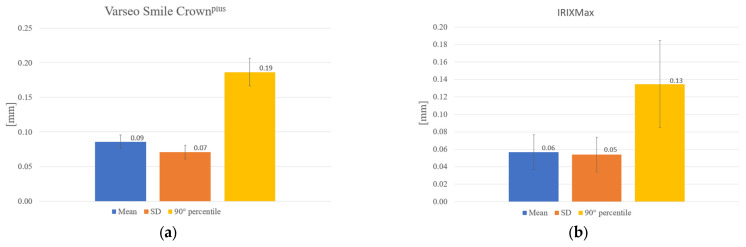
Geometric deviations ± standard deviations obtained in (**a**) G1 (Varseo Smile Crown^plus^), (**b**) G2 (IRIXMax).

Both tested materials demonstrated deviations that may be considered within clinically acceptable ranges for indirect restorations. However, statistically significant differences were observed between the two groups (Kolmogorov–Smirnov *p*-values equal to 0.09, 0.05 and 0.09, respectively, for mean, standard deviation and 90th percentile distributions across samples, according to Figure 3a,b. Within the resin-based group, SLA-printed IRIXMax demonstrated superior geometric fidelity compared to DLP-printed Varseo Smile Crown^plus^. Specifically, IRIXMax showed a lower mean deviation (≈0.06 mm) compared to Varseo Smile Crown^plus^ (≈0.09 mm) and a smaller standard deviation (0.05 mm vs. 0.07 mm) and a reduced 90th percentile value (0.13 mm vs. 0.19 mm), indicating a more accurate reproduction of the original STL model. The distribution of deviations for IRIXMax appeared more homogeneous among specimens, whereas Varseo Smile Crown^plus^ showed greater variability, particularly along the internal axial walls. Although standard deviation values were of similar magnitude to the mean deviations in both groups, suggesting localized point-to-point variability, the overall geometric fidelity of IRIXMax was significantly higher. The null hypothesis was therefore rejected.

### 3.2. Deviation Analysis Distribution

**Figure 4:**
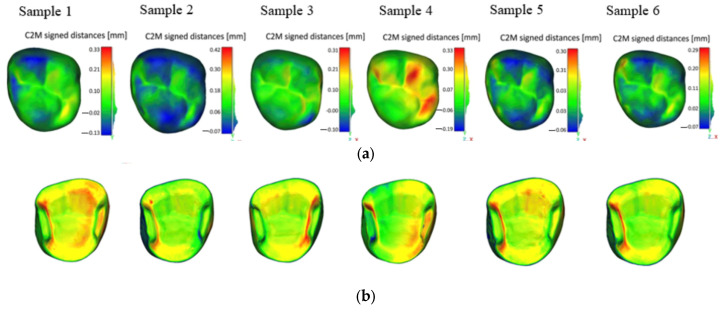
Spatial distribution of errors for Varseo Smile Crown^plus^ samples: (**a**) occlusal surface; (**b**) internal surface.

**Figure 5:**
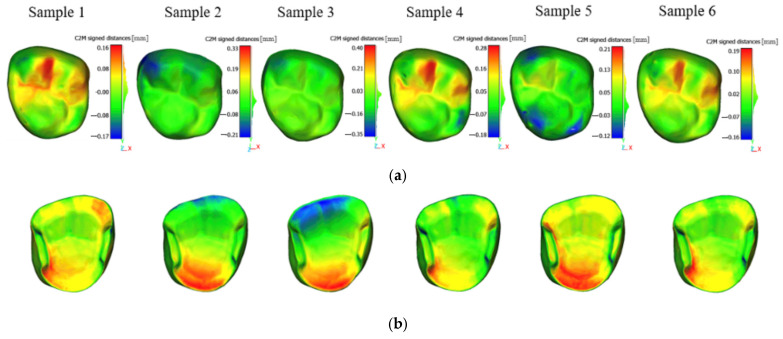
Spatial distribution of errors for IRIXMax samples: (**a**) occlusal surface; (**b**) internal surface.

A spatial distribution analysis of the geometric error was conducted by generating deviation color maps, as illustrated in the following figures (Figure 4 and Figure 5). These maps allowed a visual localization of the main deviations across both the occlusal and the internal surface. For Varseo Smile Crown^plus^ samples (Figure 4), the deviation maps revealed a homogeneous and repeatable error distribution among all seven specimens, with the most critical regions consistently located along the internal axial walls in contact with the tooth stump (Figure 4a,b). The IRIXMax material exhibited a different error pattern, showing localized deviations on both the occlusal and internal surfaces, with good spatial consistency in the location of critical areas across different samples (Figure 5a,b).

## 4. Discussion

This study assessed the geometric fidelity of 3D-printed resin composite overlays intended for adhesive indirect restorations, comparing specimens fabricated via stereolithography with IRIXMax resin and digital light processing with Varseo Smile Crown^plus^ against their original STL reference model. Rather than examining the mechanical properties of the materials, the investigation focused on the precision of the two 3D printing systems, each paired with its proprietary resin, in replicating the CAD-designed morphology of the overlay as faithfully as possible. The results revealed substantial differences in dimensional accuracy between the printing technologies, necessitating rejection of the null hypothesis.

Among the tested technologies and materials, IRIXMax printed via SLA exhibited superior geometric accuracy, reflected by lower mean deviations and reduced 90th percentile values compared with Varseo Smile Crown^plus^, printed using DLP. This outcome aligns with previous studies reporting higher 3D-printing performances of SLA-printed resin composites due to more homogeneous polymerization, finer resolution, and reduced layer-to-layer scattering [16,17,24]. Spatial deviation maps confirmed that distortions in SLA-printed crowns were localized and symmetrically distributed, indicating predictable behavior and consistent process repeatability. SLA technology, with its precise light projection and controlled resin curing, inherently offers advantages in achieving finer details and smoother surfaces [25]. In contrast, DLP-printed Varseo Smile Crown^plus^ samples showed greater variability in geometric deviations. Systematic positive deviations were observed along internal vertical surfaces, likely resulting from polymerization shrinkage, anisotropic post-curing deformation, and mechanical removal of supports. These results suggest that material-specific process optimization is essential even within composites designed for definite restorations [12,13,26,27,28]. The larger pixel size and volumetric light exposure inherent in DLP technology can lead to increased scattering and reduced resolution, contributing to the observed larger and more widespread deviations in Varseo Smile Crown^plus^ samples [29]. The comparative analysis underscores that differences in printing technology, compounded by material composition, significantly influenced geometric accuracy. Trueness, the standard metric for assessing conformity to the original STL file, is commonly expressed as root mean square deviation following superimposition of the scanned restoration onto the reference CAD/STL model [29]. The dimensional accuracy reported for milled crowns varies substantially depending on factors such as material composition, milling machine specifications, tool diameter, sintering protocols, and the specific surface regions analyzed [30,31,32]. One study reported a trueness of 61 ± 22 µm for inner surfaces and 55 ± 18 µm for occlusal surfaces of restorations for milled restorations [33]. In the present study, while both materials fall within clinically acceptable ranges for single-unit restorations (generally reported to be below 100–120 µm), the SLA-printed crowns demonstrated more consistent adherence to the STL reference geometry, highlighting the advantage of SLA when precise fit and minimal chairside adjustment are required [31]. This finding might be related both to material composition and to 3D printing technologies; nonetheless, the relevance of the second factor is supported by recent in vitro studies showing statistically significant differences in marginal gaps between SLA- and DLP-printed crowns, favoring SLA for definitive applications [16,17,24,34].

A more comprehensive materials science explanation for the observed differences between SLA and DLP restorations involves the interplay between polymerization kinetics, degree of conversion (DC), crosslink density, and volumetric shrinkage, all of which directly affect dimensional stability in methacrylate-based dental resins. In VPP systems, light exposure initiates radical polymerization, but the spatiotemporal distribution of energy differs substantially between laser-scanning SLA and layer-projection DLP technologies. SLA employs a focused laser beam that sequentially scans each cross-section, allowing localized and progressive network formation. This stepwise curing strategy may promote more gradual gelation and vitrification, potentially enabling partial stress relaxation before subsequent layers are deposited, thereby limiting cumulative distortion [1,27,35]. Conversely, DLP systems polymerize an entire layer simultaneously through patterned light projection. This results in rapid volumetric gelation across a broad surface area, reducing the time available for viscous flow and stress dissipation prior to network vitrification. Experimental studies on 3D-printed dental resins demonstrated that higher or spatially heterogeneous DC values can be associated with increased crosslink density and greater volumetric shrinkage, which in turn correlates with dimensional deviation from the original CAD geometry [8,36]. Furthermore, research evaluating shrinkage behavior in photopolymer resins has confirmed a direct relationship between volumetric contraction and geometric inaccuracy in 3D-printed parts, irrespective of the hardware used, underscoring shrinkage as a primary determinant of trueness [27,37].

In addition, DLP technology relies on fixed pixel architecture to define each voxel, and light scattering at pixel boundaries may contribute to edge overcuring and conversion gradients across the layer thickness. Such spatial heterogeneity in polymer network formation can generate anisotropic shrinkage vectors, particularly along vertical internal surfaces, consistent with the systematic positive deviations observed in the present DLP group. By contrast, the continuous vector tracing characteristic of SLA may reduce voxel boundary effects and promote smoother contour interpolation, thereby enhancing dimensional predictability relative to the STL reference [38,39].

Collectively, differences in energy delivery mode, degree of conversion distribution, crosslink density evolution, and shrinkage-induced residual stress accumulation provide a plausible mechanistic rationale for the superior trueness observed in SLA-printed restorations compared with DLP-printed counterparts in this study. The discrete nature of DLP projection, wherein an entire layer is cured simultaneously, often leads to an over-curing effect at the boundaries of the illuminated regions, especially on flat surfaces, which can contribute to undesirable dimensional changes and inhomogeneous polymerization [40]. This can result in decreased dimensional accuracy and potentially affect the mechanical properties of the final product [41]. However, a limitation of this study is its lack of a comprehensive examination of the interrelationships among degree of conversion, monomer-to-polymer ratio, and mechanical properties. Future investigations could delve into these aspects, utilizing advanced spectroscopic and mechanical testing techniques to elucidate the precise mechanisms underlying the observed differences in performance between SLA and DLP technologies.

From a clinical perspective, restoration fit is crucial for long-term success, also in terms of material wear and fatigue resistance [42,43]. Poor marginal or internal adaptation can compromise adhesive bonding, increase the risk of secondary caries, and contribute to mechanical or biological failures [30,44]. The predictable accuracy of SLA-printed IRIXMax crowns suggests that this material and technology combination may be preferable in situations requiring minimal adjustment, limited cement space and high accuracy.

Despite these promising results, some limitations should be acknowledged. Firstly, the study evaluated a single standardized geometry, which may not fully represent the complexity of clinical tooth preparations. Secondly, the use of an intraoral scanner for post-processing evaluation, although reflective of clinical practice, may have lower resolution than laboratory-based metrology systems [45]. Thirdly, support design, build orientation, and post-curing protocols were standardized but not individually optimized for each material, potentially affecting localized deviations. Finally, considered samples differed both for material composition and the 3D printing technologies; therefore, the influence of each factor cannot be isolated at the current stage. Future research should address this issue. In addition, it should include a wider variety of tooth geometries, integrate mechanical [6] and biomechanical [32] performance metrics, and investigate the interaction between support configuration and spatial distortion patterns.

## 5. Conclusions

This investigation demonstrates that composite restorations fabricated via stereolithography exhibited superior dimensional accuracy relative to those produced by digital light processing, thereby conferring a clinical advantage for manufacturing single-unit adhesive restorations. A systematic assessment of geometric fidelity, coupled with process optimization, is imperative to achieve reproducible outcomes in additively manufactured dental prostheses. Incorporating high-resolution deviation mapping, material-tailored curing protocols, and technology-specific printing parameters will be essential for formulating robust, evidence-based guidelines in the additive manufacturing of permanent restorations. Further studies should also encompass a broader spectrum of materials and technologies, alongside various scanning and printing methods, to enhance the reliability and generalizability of these findings. Additionally, investigations into varying support structure configurations and post-processing procedures are warranted to ascertain their impact on print accuracy, efficiency, and material utilization.

## Figures and Tables

**Table 1:** Investigated materials and manufacturer information.

Nomenclature	Material	Manufacturer	Solid Loading (wt%)	Filler	3D-Printing Technology
G1	Varseo Smile Crown^plus^	BEGO, Bremen, Germany	30–50; 33.3	Silanized dental glass (particle size 0.7 μm)	DLP
G2	IRIXMax	DWS S.r.l, Thiene, Italy	42	Ceramic spheric inclusions (approximately ranging between 1 and 8 µm in diameter)	SLA

## Data Availability

The original contributions presented in this study are included in the article. Further inquiries can be directed to the corresponding author.

## Abbreviations

The following abbreviations are used in this manuscript:

3D	Three-dimensional
CAD	Computer-Aided Design
CAM	Computer-Aided Manufacturing
STL	Standard Tessellation Language
VPP	Vat-photopolymerization
DLP	Digital Light Processing
SLA	Stereolithography
TSLA	Tilting Stereolithography
CMM	Coordinate Measuring Machine
micro-CT	micro-Computed Tomography
CEJ	Cemento-Enamel Junction
ICP	Iterative Closest Point
RMS	Root Mean Square
SD	Standard Deviation
DC	Degree of Conversion
